# Correction: Harshbarger et al. Structures of the Varicella Zoster Virus Glycoprotein E and Epitope Mapping of Vaccine-Elicited Antibodies. *Vaccines* 2024, *12*, 1111

**DOI:** 10.3390/vaccines14090746

**Published:** 2026-08-28

**Authors:** Wayne D. Harshbarger, Genevieve Holzapfel, Nishat Seraj, Sai Tian, Chelsy Chesterman, Zongming Fu, Yan Pan, Claire Harelson, Dongjun Peng, Ying Huang, Sumana Chandramouli, Enrico Malito, Matthew James Bottomley, James Williams

**Affiliations:** 1 GSK, Rockville, MD 20850, USA; genevieve.holzapfel@nih.gov (G.H.); natasha.seraj@astrazeneca.com (N.S.); sai.x.tian@gsk.com (S.T.); chelsy.c.chesterman@gsk.com (C.C.); zongming.x.fu@gsk.com (Z.F.); yan.x.pan@gsk.com (Y.P.); charels1@alumni.jh.edu (C.H.); dpeng@its.jnj.com (D.P.); ying.huang@wuxibiologics.com (Y.H.); sumana.chandramouli@modernatx.com (S.C.); enrico.malito@schrodinger.com (E.M.); matthewjamesbottomley@gmail.com (M.J.B.); 2 WuXi Biologics, Cranbury, NJ 08512, USA; 3 Moderna Therapeutics Inc., Cambridge, MA 02142, USA; 4 Schrödinger, Inc., New York City, NY 10036, USA; 5 Dynavax Technologies Corporation, Emeryville, CA 94608, USA

The authors would like to make the following corrections to this published paper [1]. In the original publication, the antibody designation 1E12 was used in error. The correct designation is 1E8. All instances of “1E12” should be replaced with “1E8”. This correction affects the following locations in the article:

Section 2.10: title and second paragraph;

Section 3.6: title and all four paragraphs;

Section 3.7: second paragraph;

Section 4 Discussion: third paragraph;

Figures 4 and 5;

Supplementary Materials paragraph;

Supplementary Figures S7–S9 and the header of the rightmost column in Table S1; 

Data Availability Statement.

No other changes have been made to the affected text, figures, tables, or Supplementary Materials. The corrected Figure 4 and Figure 5 are as follows. The corrected Supplementary Materials are as attached.

The authors state that the scientific conclusions are unaffected. These corrections were approved by the Academic Editor. The original publication has also been updated.

## Figures and Tables

**Figure 4:**
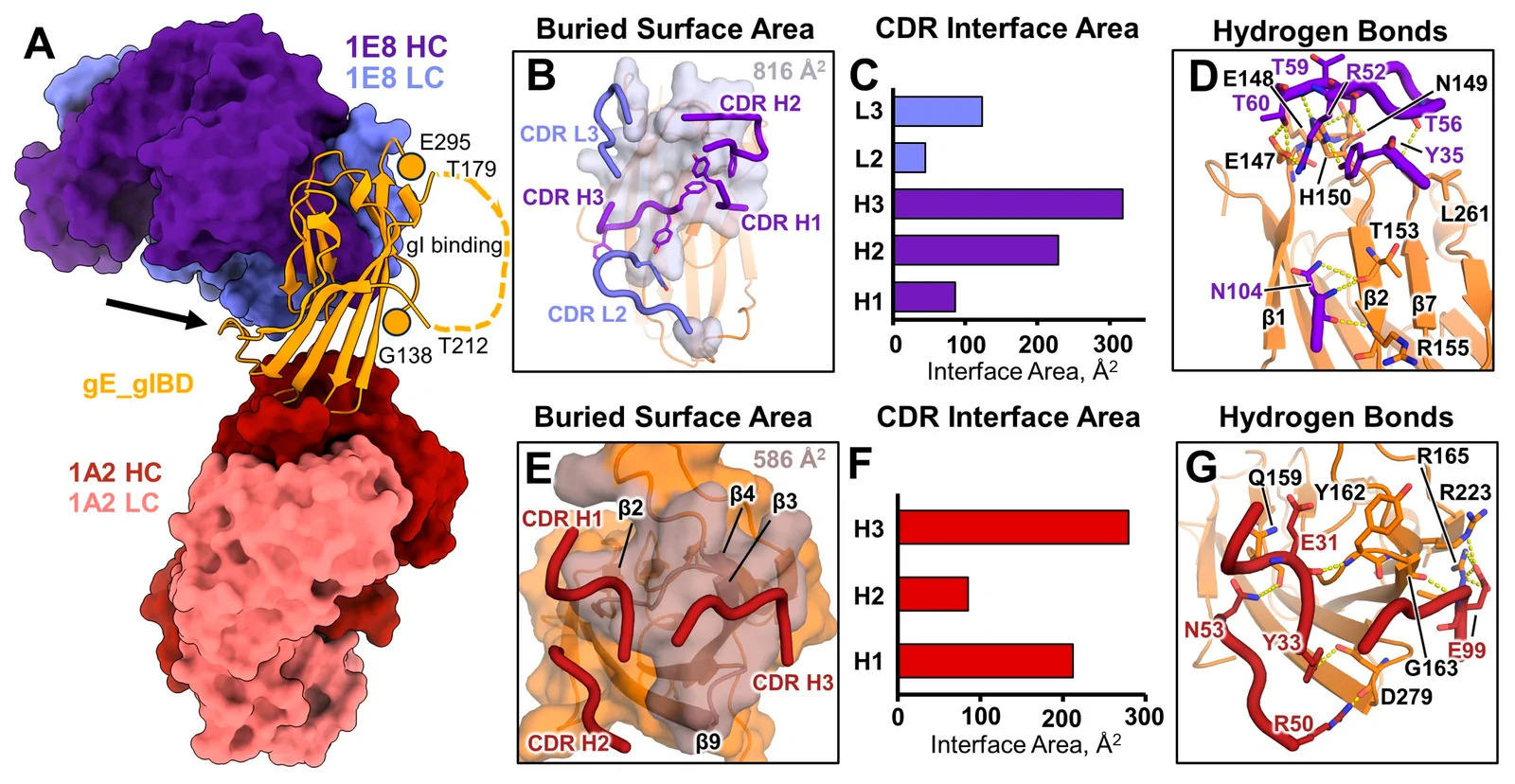
Epitope mapping of the gE_gIBD. (**A**) X-ray structure of the gE_gIBD in ternary complex with Fabs 1A2 and 1E8, which are shown in surface and colored as labeled in the panel. The black arrow indicates a loop on gE_gIBD, which was not resolved in the 1A2-bound structure. The residues forming the gIBD are depicted with a dashed yellow line. (**B**) The 1E8 CDRs that contact gE are shown in cartoon tubes and sticks and the BSA on gE is shown in transparent surface. (**C**) Graph indicating the 1E8 CDR interface areas on gE. (**D**) Hydrogen bond interactions between gE and Fab 1E8. (**E**) The 1A2 CDRs that bury surface on gE are shown as cartoon tubes and gE is shown in transparent surface with the regions buried by 1A2 shaded dark. (**F**) Graph indicating the 1A2 CDR interface areas on gE. (**G**) Hydrogen bond interactions between gE and Fab 1A2.

**Figure 5:**
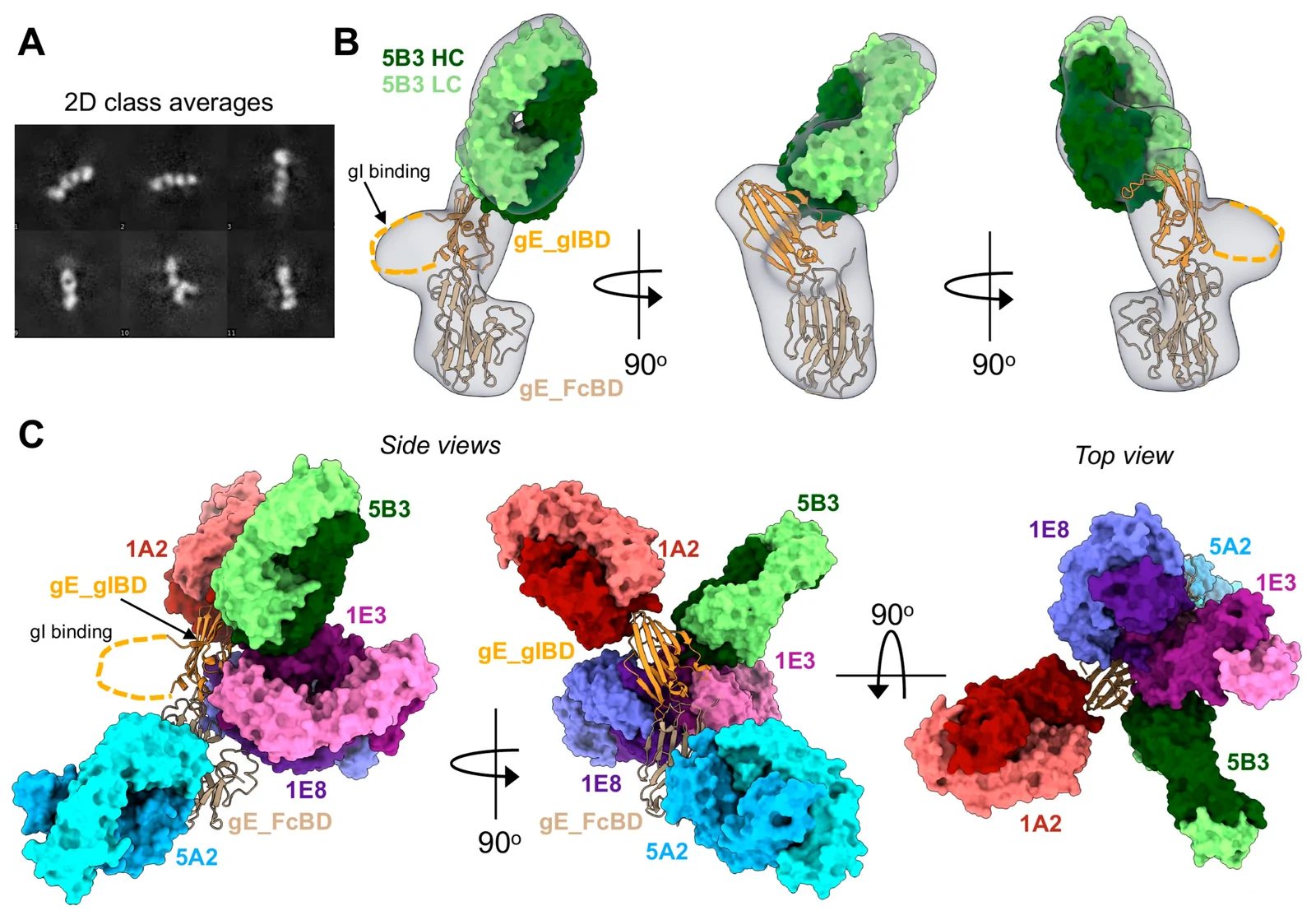
Cryo-EM analysis of gE_FL and epitope mapping of Fab 5B3. (**A**) Cryo-EM 2D classes for FL gE in complex with Fab 5B3. (**B**) Low-resolution 3D reconstruction of the FL gE:5B3 Fab complex. The gE_gIBD and gE_FcBD X-ray structures, and a 5B3 homology model were fitted in the EM density. Unfit density is presumed to be the gIBD and is indicated with a dotted orange line. The disordered N-terminal domain was not visible. (**C**) The fitted structures from (**B**) docked with the 5A2, 1E3, 1E8, and 1A2 Fabs to their respective epitopes. The middle view best displays how one face of gE is not recognized by the Abs used in this study.
