# Supplementary material for: Structures of the Varicella Zoster Virus Glycoprotein E and Epitope Mapping of Vaccine-Elicited Antibodies
Source: Vaccines (Basel). 2024 Sep 27;12(10):1111. doi: 10.3390/vaccines12101111 (PMC11511291; doi:10.3390/vaccines12101111)
Supplement: Supplementary file 1 [file vaccines-12-01111-s001.zip › vaccines-3190949-supplementary.pdf]

Supplementary Materials for

Structure of the varicella zoster virus glycoprotein E and epitope mapping of vaccine elicited antibodies

Wayne D. Harshbarger, *et al.*

*Corresponding author: Wayne Harshbarger, wayne.harshba@gmail.com

This PDF file includes:

Figures S1 to
S9 Tables S1
to S2

Other Supplementary Materials for this manuscript include the following:

Data S1

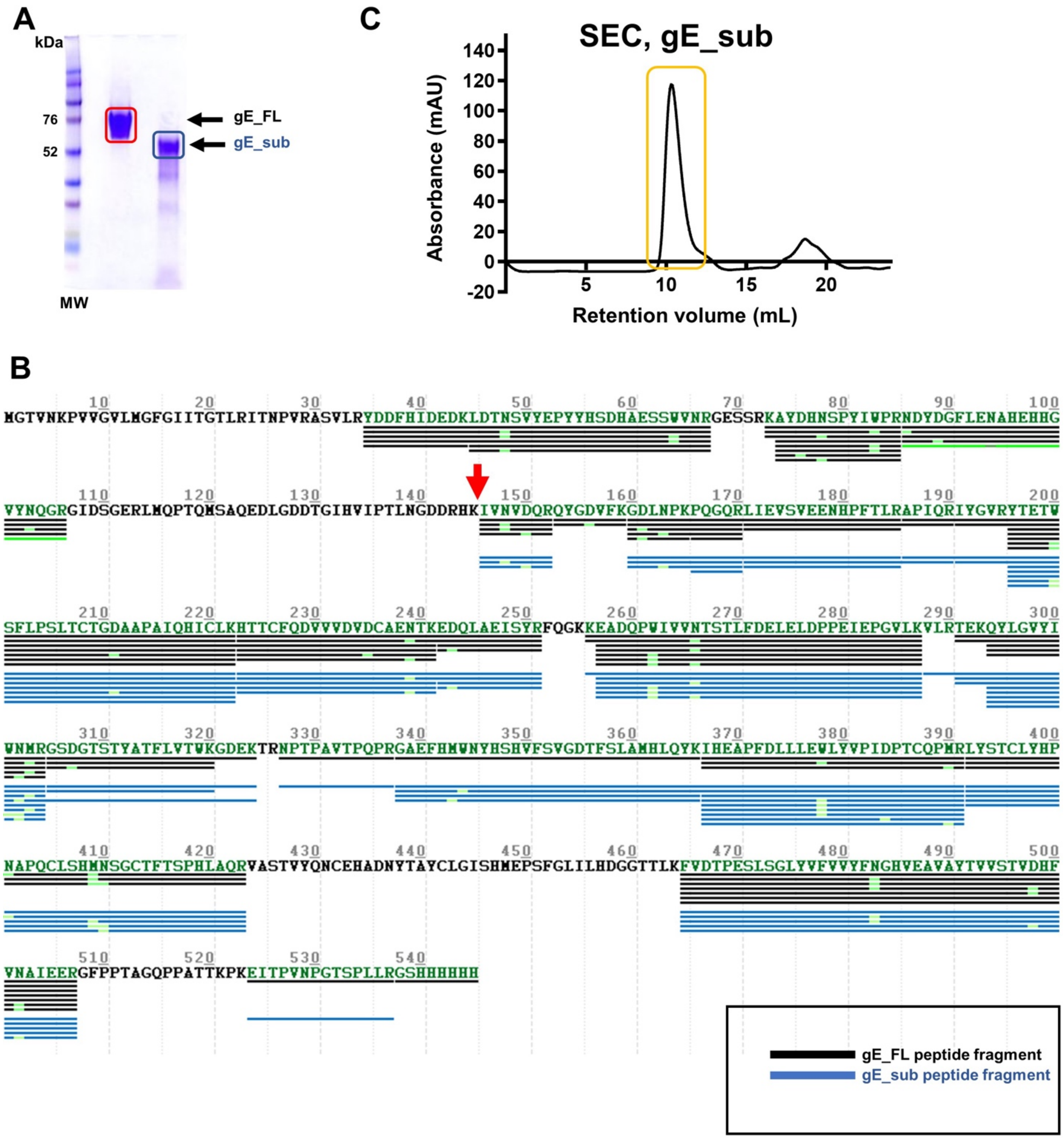

Figuse S1. Analysis of proteolytically cleaved gE_FL. (**A**) SDS-PAGE gel for gE_FL and the subtilisin cleaved gE. (**B**) LC-MS/MS fragments from analysis of gE_FL and subtilisin cleaved gE_FL. The red arrow indicates the potential cleavage spot which was selected for making the gE_sub construct. (**C**) SEC chromatogram for the recombinantly produced gE_sub.

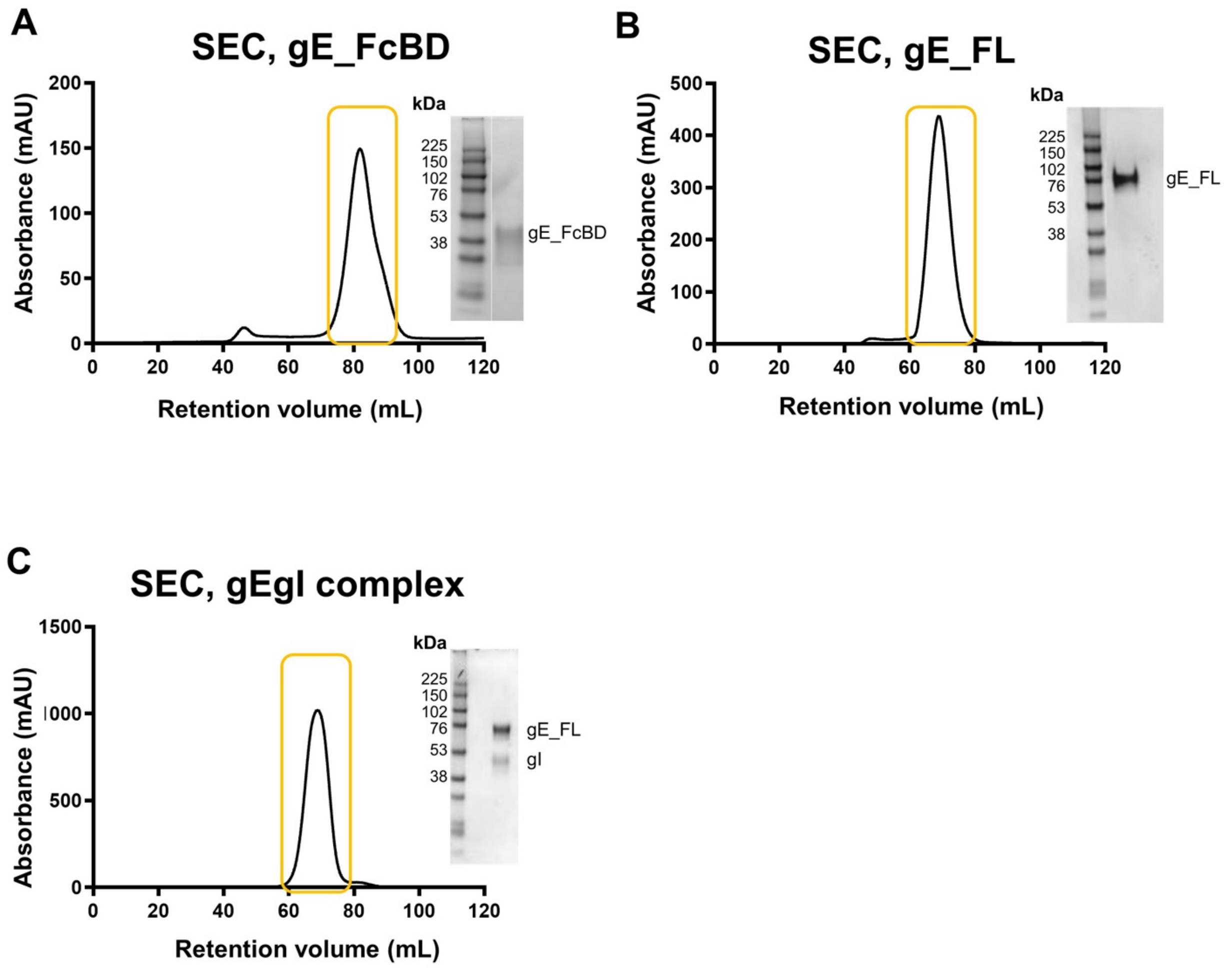

Figure S2. Recombinantly produced gE constructs. SEC chromatograms and SDS_PAGE gels for (**A**) gE_FcBD; (**B**) gE_FL; and (**C**) gEgI heterodimer.

A

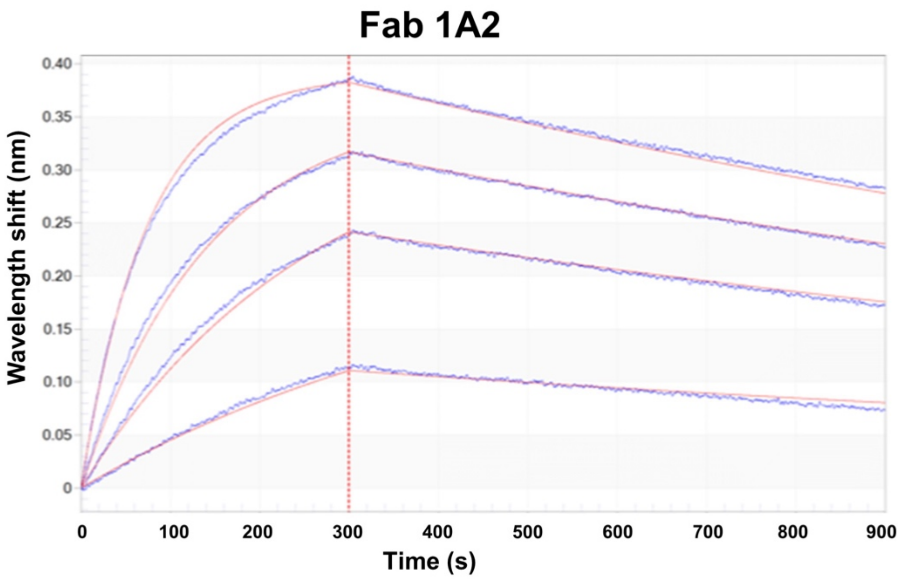
Fab 1A2
Wavelength shift (nm)
0.40
0.35
0.30
0.25
0.20
0.15
0.10
0.05
0
0
100
200
300
400
500
600
700
800
900
Time (s)

B

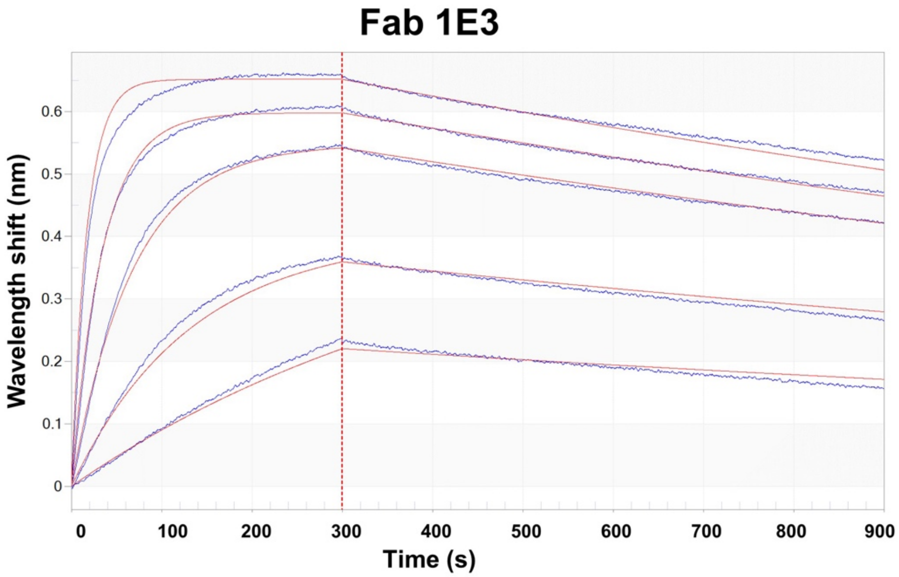
Fab 1E3
Wavelength shift (nm)
0.6
0.5
0.4
0.3
0.2
0.1
0
0
100
200
300
400
500
600
700
800
900
Time (s)

C

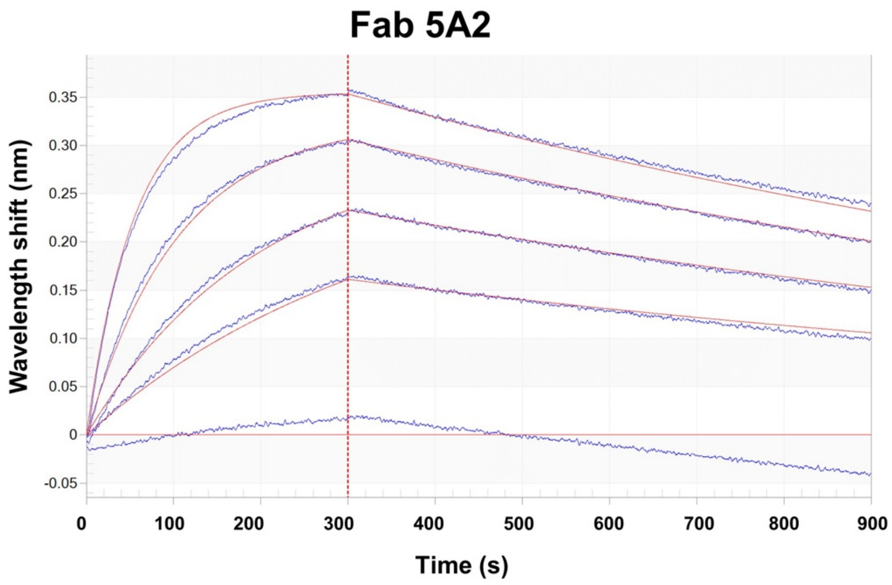

D

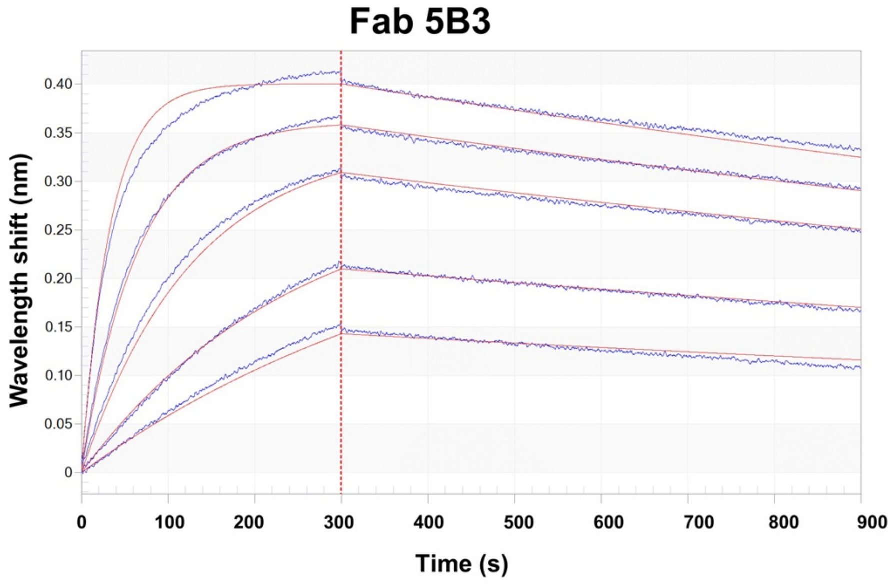

Figure S3. Binding kinetics for anti-gE Fabs. BLI curves for (**A**) Fab 1A2; (**B**) Fab 1E3; (**C**) Fab 5A2; and (**D**) Fab 5B3 to gE_FL. All Fabs showed nM binding affinity (Table S2). Concentrations ranged from 100 nM to 3.12 nM for Fab 1E3 and from 50 nM to 3.12 nM for the other Fabs.

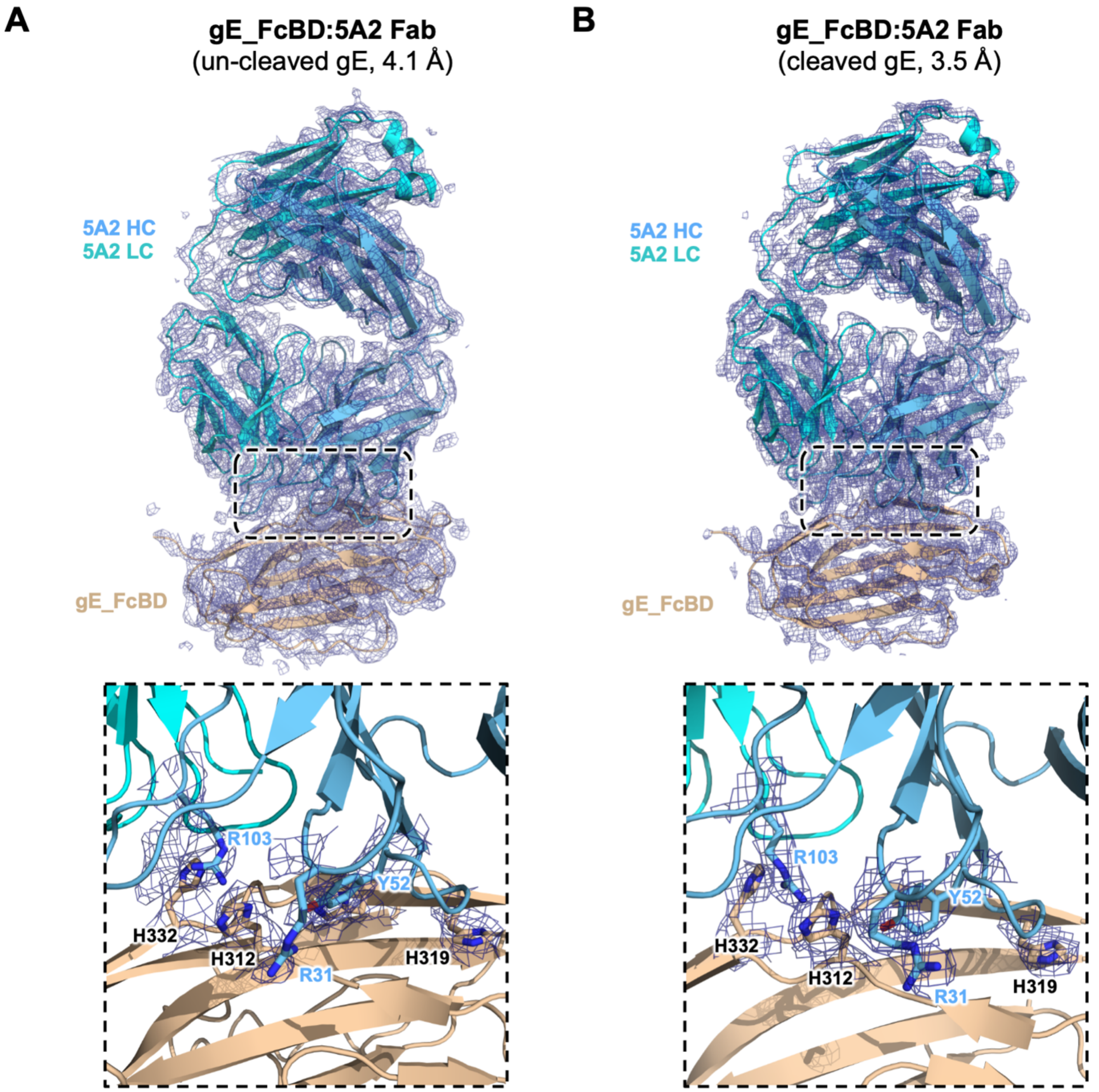

Figure S4. Electron density for gE_FcBD:5A2 Fab complexes solved by x-ray crystallography. (**A**) Density for the 4.1 Å resolution structure and zoomed in view of the paratope:epitope interface. (**B**) Density for the 3.5 Å resolution structure and zoomed in view of the

paratope:epitope interface. The gE_Fc_BD:5A2 Fab complex is shown in cartoon representation. The 2Fo-Fc electron density maps are scaled to 1σ and shown as dark blue mesh. Representative side chains at the interface are shown as sticks.

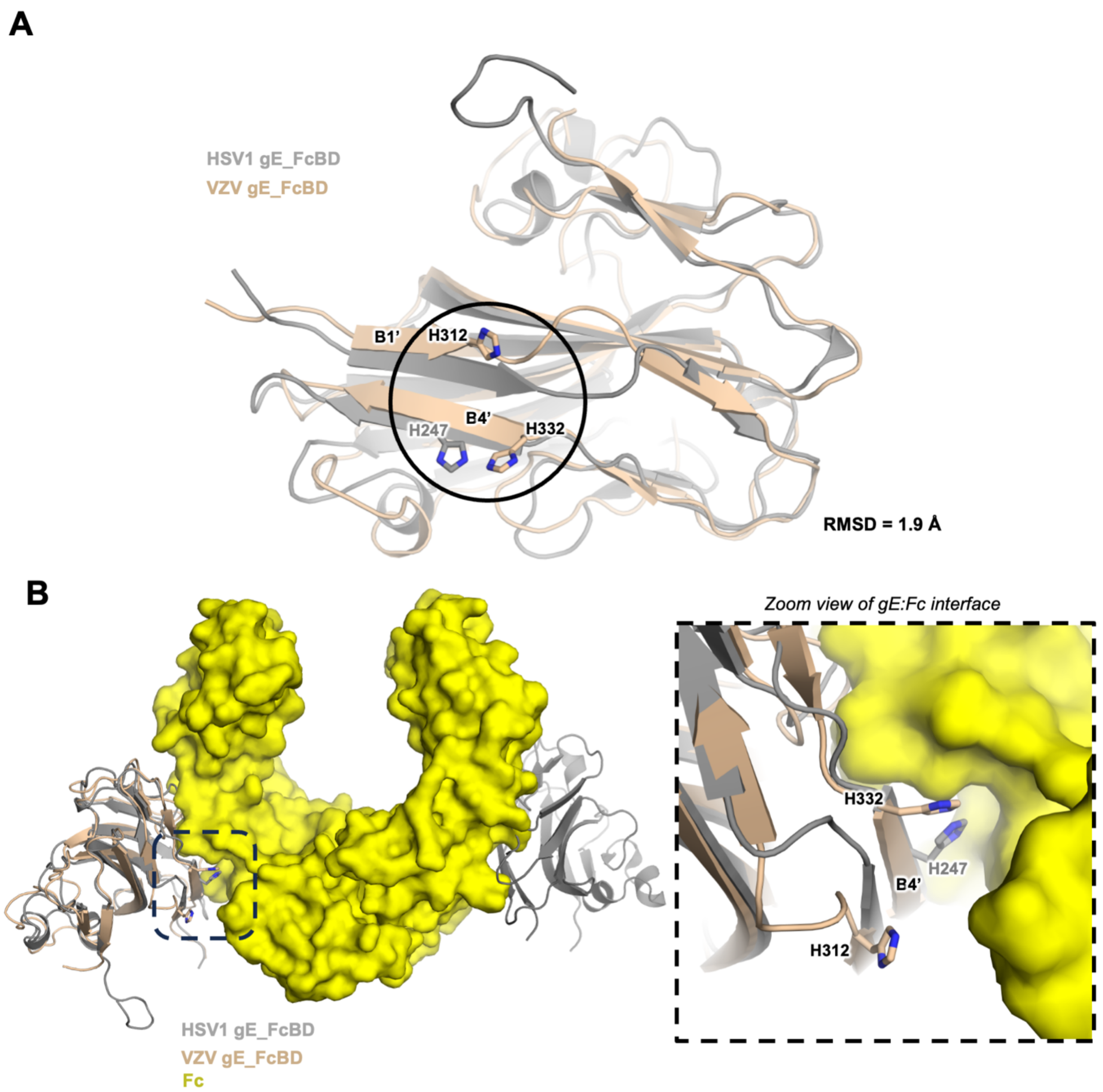

Figure S5. Comparison of gE_FcBD with HSV1 gE and Fc docking. (A) Superposition of VZV gE_FcBD with the corresponding HSV1 gE structure (PDB 2GIY). The black circle indicates the location for histidine residues that are in proximity between the two structures and predicted to be at the interface with Fc binding. (**B**) Superposition of VZV gE_FcBD with the HSV1 gE in complex with Fc (PDB 2GJ7). Zoom window shows the interface and location of histidine residues. Each gE is shown in cartoon representation and Fc is shown as surface and colored yellow. Histidine residues are shown as sticks.

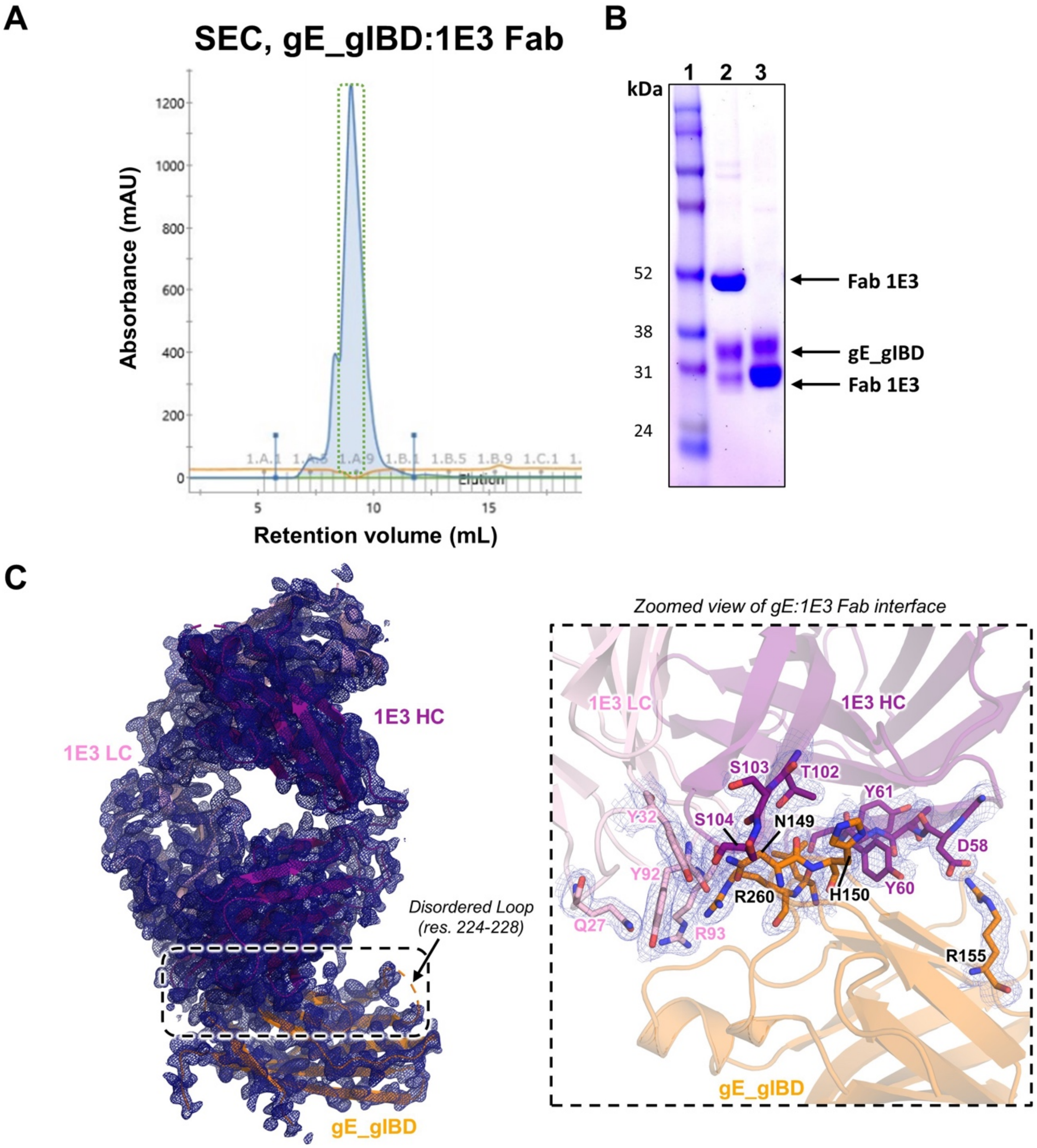

Figure S6. Purification and electron density for the gE_gIBD:1E3 Fab complex. (A) SEC and (B) SDS-PAGE analysis for purified gE_gIBD:1E3 Fab complex. (C) Structure shown with 2Fo-Fc electron density map scaled to 1σ shown as dark blue mesh. Zoom window shows interface residues in stick representation with corresponding electron densities.

Primary Ab \ Secondary Ab	1E3	1E12	1D7	1A2	5A2
1E3	-	-	-	+	+
1E12	-	-	-	+	+
1D7	-	-	-	+/-	+
1A2	+	+	+/-	-	+
5A2	+	+	+	+	-

Figure S7. Epitope binning for Fabs 1E12 and 1D7 against 1E3, 1A2, and 5A2. Binning indicated that 1E12 and 1D7 recognize the gE_gIBD as they each compete for binding with Fab 1E3.

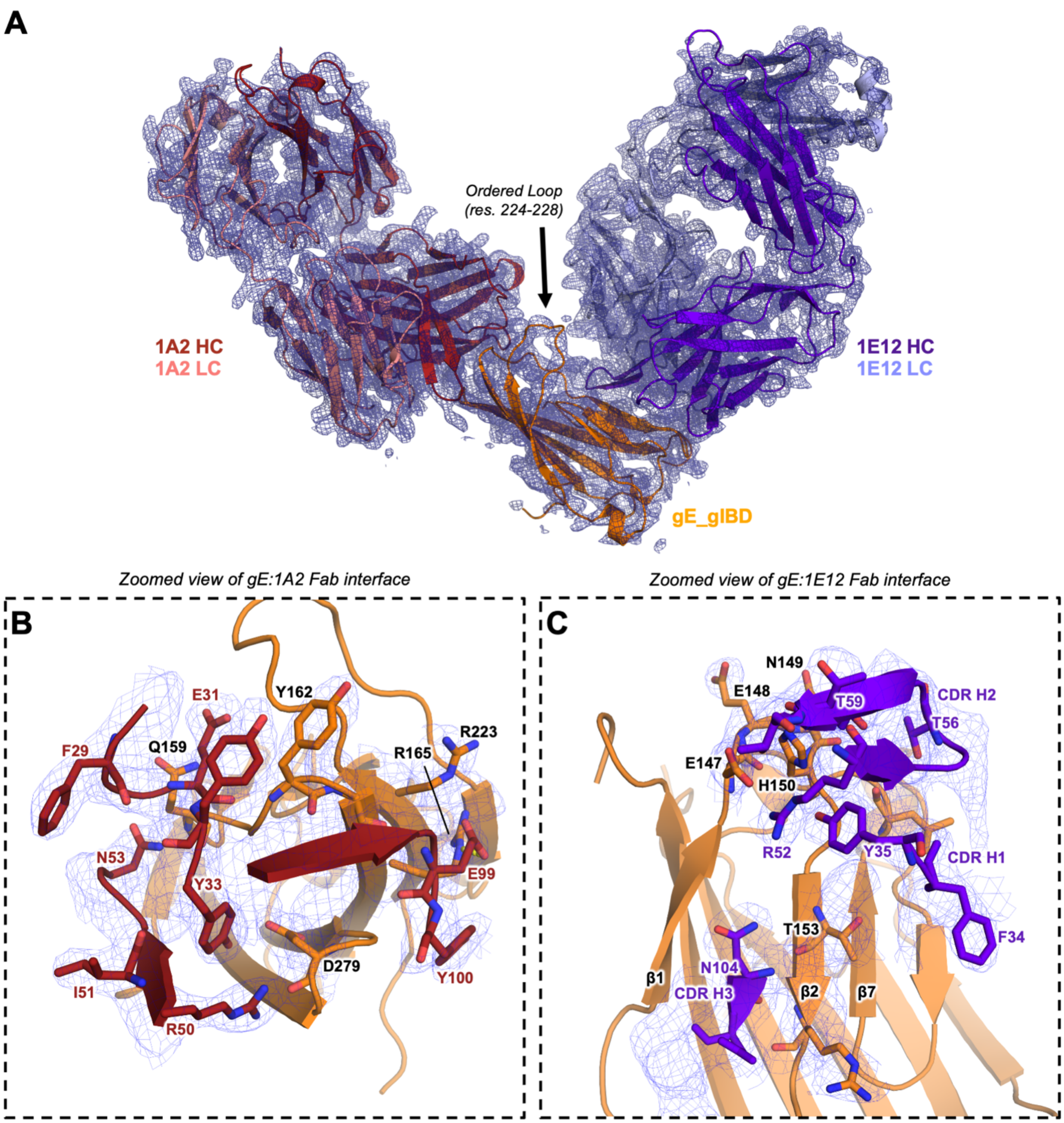

Figure S8. Electron density for gE_gIBD:1A2:1E12 ternary complex. (**A**) Entire complex is shown in cartoon representation and colored as in previous panels. Zoom view of gE_1A2 (**B**) or gE_1E12 (**C**) interface. Residues making interactions are shown as sticks. The 2Fo-Fc electron density map is scaled to 1σ and shown as dark blue mesh.

A

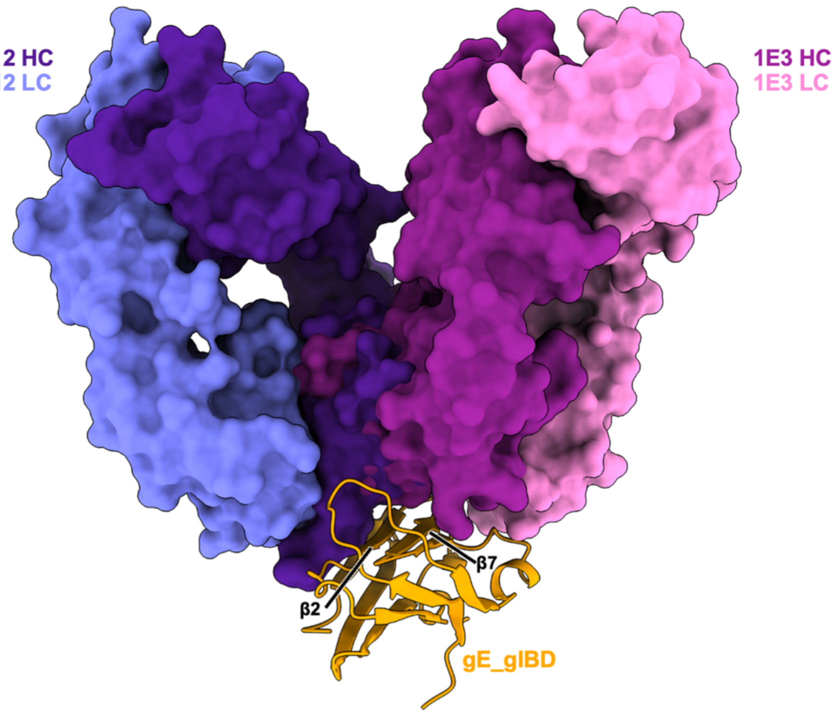

B

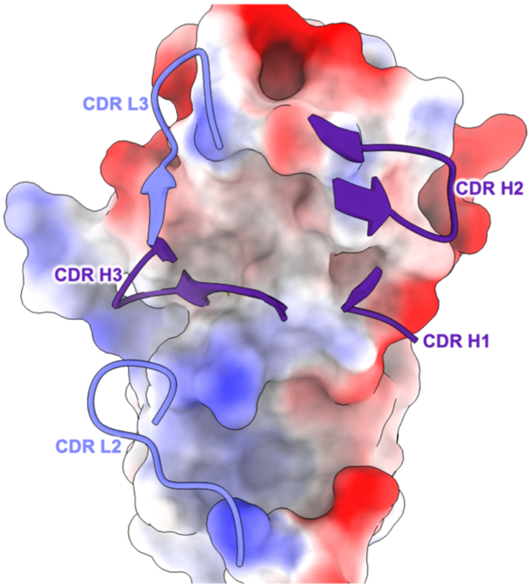

Figure S9. Epitope competition and surface charge for Fab 1E12. (**A**) Fabs 1A2 and 1E12 shown on the same molecule of gE_gIBD to illustrate clashing of the heavy chains. (**B**) The surface for gE_gIBD is shown with electrostatic potentials (red = negative, blue = positive, white = neutral). Heavy and light chain CDRs are shown as cartoon. The epitope is predominantly neutral or positively charged.

Table S1. X-ray data collection and refinement statistics

	gE_FcBD:5A2 Fab	gE_FcBD:5A2 Fab (cleaved)	gE_gIBD:1E3 Fab	gE_gIBD:1E12:1A2 Ternary Complex
**Data Collection**				
Wavelength (λ)	1	1	1	1
Resolution range (Å)	45.7-4.33 (4.48-4.33) *	38.13-3.53 (3.66-3.53) *	30.39-1.90 (1.97-1.90) *	47.51-3.09 (3.20-3.09) *
Space group	$P4_12_12$	$P2_1$	$C222_1$	C2
Cell dimensions				
a, b, c (Å)	274.8, 274.8, 129.0	105.1, 60.4, 114.7	44.6, 193.7, 179.8	218.8, 63.5, 96.5
α, β, γ (°)	90, 90, 90	90, 93.9, 90	90, 90, 90	90, 100, 90
Total reflections	455431 (43751)	67711 (6816)	841792 (81002)	70169 (5657)
Unique reflections	33380 (3055)	16382(1136)	61666 (5903)	23336 (1958)
Multiplicity	13.6 (13.3)	3.8 (3.9)	13.7 (13.5)	3.0 (2.7)
Completeness (%)	99.00 (93.25)	90.73 (64.11)	99.3 (96.2)	94.14 (81.72)
I/σI	9.3 (4.1)	15.2 (4.9)	8.9 (0.9)	9.5 (2.1)
Wilson B-factor	46.59	53.03	30.61	59.18
R-merge	0.571 (1.64)	0.209 (0.609)	0.182 (2.310)	0.148 (0.382)
$CC_{1/2}$	0.94 (0.70)	0.96 (0.72)	0.99 (0.72)	0.95 (0.73)
**Refinement**				
Resolution (Å)	45.7-4.3	38.13-3.53	30.39-1.90	47.51-3.09
No. reflections	33098	16380	61389	22855
R_{work} / R_{free} (%)	23/28	26/30	22/23	23/27
No. protein atoms	18372	8421	4236	7752
No. of solvent atoms	0	0	178	0
No. of ligand atoms	0	0	43	0
R.m.s. deviations:				
Bond lengths (Å)	0.012	0.003	0.012	0.003
Bond angles (°)	0.75	0.70	1.22	0.66
Ramachandran plot#				
Favored (%)	90.1	91.5	96.2	97.3
Allowed (%)	9.9	8.3	3.6	2.7
Outliers (%)	0.1	0.2	0.2	0
Average B-factor	90.8	43.8	43.7	61.4
macromolecules	90.8	43.8	43.7	61.4
Solvent	N/A	N/A	42.4	N/A
Ligands	N/A	N/A	52.5	N/A
PDB ID	8V5P	8V5S	8V5Q	8V5L

R.m.s. deviation, root-mean square deviation.
Values in parentheses are for the highest resolution shell.
#*Measured using Molprobity*

Table S2. Binding analysis of anti-gE Fabs

gE construct	Fab	k_{on} ($M^{-1}s^{-1}$)	k_{off} (s^{-1})	K_D (nM)
gE_FL	1E3	5.71×10^5	4.2×10^{-4}	0.74
	5B3	5.97×10^5	3.49×10^{-4}	0.59
	1A2	2.62×10^5	5.33×10^{-4}	2.0
	5A2	3.53×10^5	7.03×10^{-4}	2.0
gE_FcBD	5A2	7.43×10^5	4.7×10^{-4}	0.63

Data S1. (separate file)

Biolayer interferometry raw data.
